# Gene Expression Profiles Reveal Distinct Mechanisms Driving Chronic Obstructive Pulmonary Disease Exacerbations

**DOI:** 10.3390/ijms26020627

**Published:** 2025-01-13

**Authors:** Melissa Bello-Perez, Eduardo García-Pachón, Nieves Gonzalo-Jimenez, Montserrat Ruiz-García, Lucía Zamora-Molina, Carlos Baeza-Martinez, Antonio Galiana

**Affiliations:** 1 Hospital General Universitario de Elche-FISABIO, 03203 Elche, Spain; egpachon@gmail.com (E.G.-P.); gonzalo_nie@gva.es (N.G.-J.); ruiz_mongar@gva.es (M.R.-G.); lucialzjc@gmail.com (L.Z.-M.); baezamartinez.c@gmail.com (C.B.-M.); antoniogaliana1@gmail.com (A.G.); 2 Infectious Diseases Unit, Hospital General Universitario de Elche, 03203 Elche, Spain; 3 Biomedical Research Networking Center for Infectious Diseases (CIBERINFEC), Instituto de Salud Carlos III, 28029 Madrid, Spain

**Keywords:** COPD, exacerbation, transcriptomics, microbiome, gene expression profiles, biomarkers, inflammatory responses, personalized management

## Abstract

Chronic obstructive pulmonary disease (COPD) exacerbations are major contributors to morbidity and mortality, highlighting the need to better understand their molecular mechanisms to improve prevention, diagnosis, and treatment. This study investigated differential gene expression profiles and key biological processes in COPD exacerbations categorized based on sputum microbiome profiling. An observational study was performed on a cohort of 16 COPD patients, who provided blood and sputum samples during exacerbations, along with five stable-state samples as controls. Exacerbations were classified using *16S rRNA* sequencing to analyze the sputum microbiota and multiplex PCR to detect respiratory viruses. Blood transcriptomic profiling was conducted using Oxford Nanopore technology, followed by differential gene expression and pathway enrichment analyses. A total of 768 regulated genes were identified across the exacerbation groups, with 35 shared genes associated with neutrophil activation. Bacterial exacerbations activated pathways related to phagocytosis and toll-like receptor signaling, while viral exacerbations were linked to pro-inflammatory responses and mitochondrial damage. Exacerbations of unknown origin showed activation of pathways involved in protozoan defense and neutrophilic asthma. Biomarkers such as *IFITM3* and *ISG15* for bacterial exacerbations, *DEFA3* for viral, and *CD47* for unknown-origin exacerbations were identified. These findings highlight distinct transcriptomic profiles and biological pathways in COPD exacerbations, emphasizing the central role of neutrophil-driven inflammation and identifying potential biomarkers for improved differential diagnosis and personalized management.

## 1. Introduction

COPD is a progressive condition that restricts airflow in the airways, resulting from a chronic and abnormal local inflammatory response to prolonged tobacco use (>70% of cases) or exposure to environmental pollutants (≈30% of cases). Recent data indicate that COPD affects 212.3 million people worldwide and causes 3.23 million deaths annually, making it the third leading cause of death globally [1].

COPD symptoms are variable (e.g., cough, fatigue, shortness of breath) and can worsen rapidly, leading to acute exacerbation episodes. Viral infections account for approximately 50% of these exacerbations, followed by bacterial infections. Additionally, there is a subset of exacerbations of unknown etiology [2] for which no clear infectious cause has been identified. Nevertheless, regardless of the causative agent, all exacerbations trigger an uncontrolled immune response. Combined with the high levels of pre-existing inflammation in these patients, this response leads to an exaggerated reaction, resulting in airway narrowing, accelerated lung function decline, and, in some cases, death [1].

The most identified pathogens in viral exacerbations are rhinoviruses and human respiratory syncytial virus [3]. *Haemophilus influenzae* and *Streptococcus pneumoniae* are the most common bacterial agents in exacerbations of bacterial origin [4]. These bacteria often appear in the context of viral infection, further worsening the clinical picture [5]. For exacerbations of unknown origin, various hypotheses are considered, including exposure to other pathogens, such as protozoa [6] or environmental pollution [7].

The diversity of causal agents complicates the clinical management of exacerbations and highlights the urgent need for a deeper understanding of the pathobiological mechanisms that induce these episodes, which are currently poorly understood. The limited available information rarely stratifies patients according to the exacerbation’s etiology. At best, data are categorized into viral and bacterial exacerbations, leaving exacerbations of unknown causes unaddressed [7]. However, some biomarkers for the general diagnosis of exacerbations have been identified [8], and significant changes have been described in the sputum microbiota of COPD patients during infectious (bacterial or viral) exacerbations—changes not observed in exacerbations of unknown origin [9].

This study aims to identify differential gene expression profiles and regulated mechanisms in COPD patients during exacerbations, analyzing the data based on the episode’s etiological trigger (bacterial, viral, or unknown). Our findings not only deepen our understanding of the molecular pathways involved in each type of exacerbation but also represent a step toward personalized medicine in COPD management, offering potential biomarkers to refine diagnosis and treatment based on the specific etiology of each exacerbation.

## 2. Results

### 2.1. Sample Classification

This study includes 16 patients (13 male and 3 female, at birth) with a median age of 74.0 years (IQR 67–79 years). Exacerbated participants presented various severity levels: seven (43.75%) were classified as GOLD 3, five (31.25%) as GOLD 4, 2 (12.5%) as GOLD 2, and only one patient (6.25%) as GOLD 1. In addition, the blood leukocyte count was 8825 cells/µL (IQR 7973–9443 cells/μL), the blood neutrophil count (BNC) was 6245 cells/µL (IQR 4695–6918 cells/μL), the lymphocyte count was 1275 cells/µL (IQR 758–1965 cells/µL), and the blood eosinophil count (BEO) was 135 cells/µL (IQR 65–258 cells/μL). The neutrophil to lymphocyte ratio (NLR) was >4 (Table 1). None of the participants died.

**Figure 1:**
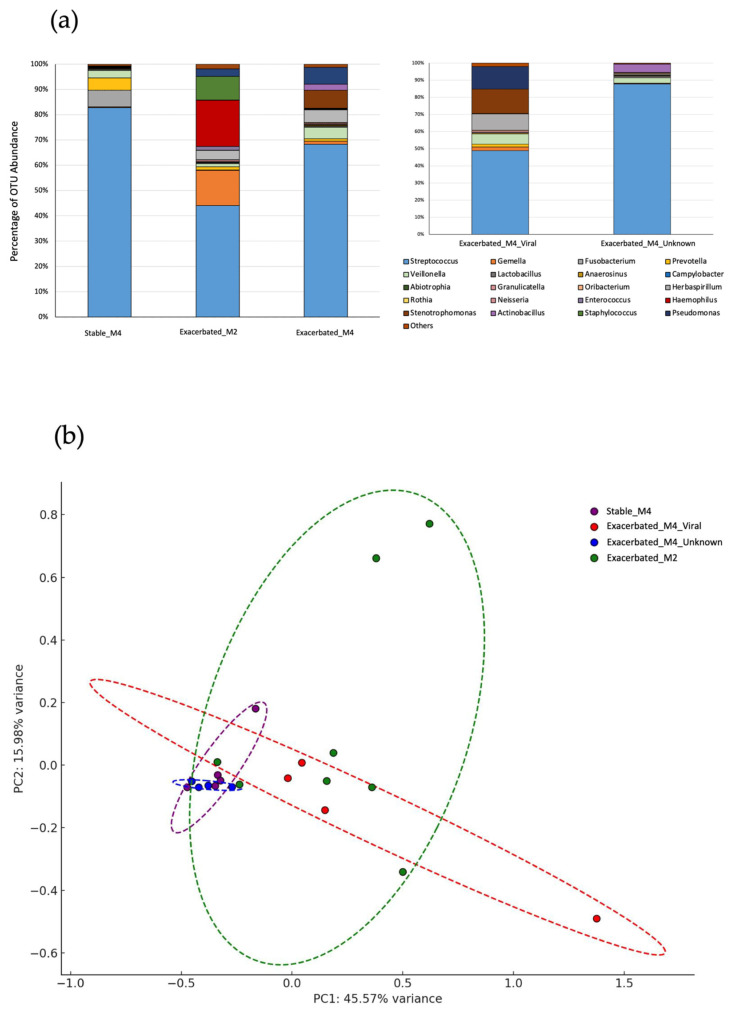
Classification of sputum samples into groups according to microbiota composition. (**a**) Relative abundance of Operational Taxonomic Units (OTUs) across clinical groups. The stacked bar plot shows the mean relative abundance at the bacterial genus level in participants. (**b**) Principal Component Analysis (PCA) of UniFrac Weighted Distance Matrix of samples by sample groups. Each sample represents an individual sample, color-coded by group: Stable_M4 (purple), Exacerbated_M2 (green), Exacerbated_M4_Viral (red), and Exacerbated_M4_Unknown (blue). Ellipses represent 95% confidence intervals for each group, indicating the variation within each sample category.

Sputum samples from stable patients primarily showed the Streptococcus genus, along with other bacterial genera such as *Prevotella*, *Fusobacterium*, and *Veillonella* (microbiome profile described previously [9] as Stable_M4). Patients with exacerbation who showed a microbiota similar to the stable group but also had respiratory virus infections, such as rhinovirus (3 samples) and metapneumovirus (1 sample), were classified as the viral exacerbation group (microbiota M4 + respiratory virus; *n* = 4, with the label Exacerbated_M4_Viral used hereafter in this text and figures). Patients in whom the Streptococcus genus represented less than 50% of the bacterial community and who showed considerable proportions of other bacterial genera (*Haemophilus*, *Gemella, Staphylococcus*, etc.) were classified as the bacterial exacerbation group (M2, *n* = 4, with the label Exacerbated_M2 used hereafter in this text and figures). Finally, patients who presented a microbiota similar to stable patients and showed no respiratory virus infection were considered the unknown cause exacerbation group (M4, *n* = 4, with the label Exacerbated_M4_Unknown used hereafter in this text and figures) (Table 1 and Figure 1).

### 2.2. Identification of Differentially Expressed Genes

Differential expression analysis in the blood of patients identified a total of 10,958 genes with expression differences between exacerbated and stable groups. A total of 76 genes were significantly down-regulated, while 692 were significantly up-regulated (*p* < 0.05). Additionally, 10,190 genes showed no significant regulation in expression levels during exacerbation (Supplementary Figure S1). The group with the highest number of differentially expressed genes compared to the stable group was the sample Exacerbated_M2 group (*n* = 292), followed by the Exacerbated_M4_Unknown group (*n* = 234) and the Exacerbated_M4_Viral group (*n* = 193) (Figure S1).

**Figure 2:**
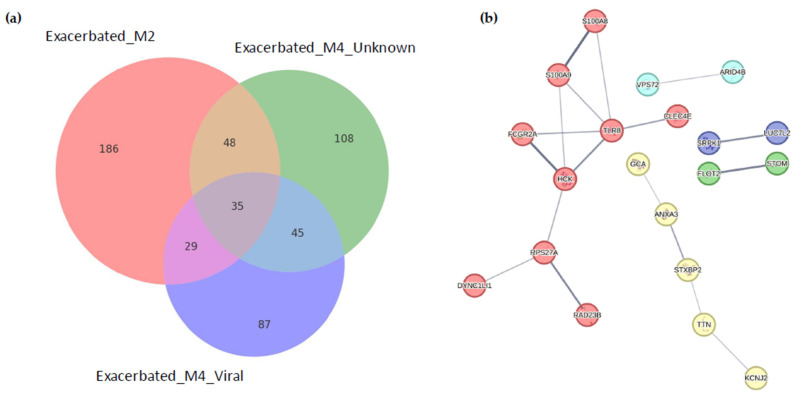
Identification of differential gene expression in patients with COPD exacerbations. (**a**) Venn diagram of significant genes expressed across clinical groups. Each circle represents the set of significant genes for one group, with unique and overlapping regions showing the distribution of shared and distinct genes. Numbers within each section denote the count of significant genes specific to each group or shared across multiple groups. (**b**) Interaction network of significant genes across clinical groups. Nodes represent genes, color-coded by functional clusters, while edges indicate known interactions based on data from the STRING database. Distinct clusters indicate functional modules, with certain genes showing connectivity across multiple groups.

A total of 35 genes were differentially expressed in three of the exacerbated groups (Figure 2a, Table 2 and Supplementary Table S1). These genes belong to five clusters (Figure 2b), with two of them involved in the activation of neutrophils within an immune response context. The rest of the genes only showed differential expression in a specific exacerbation group. A total of 186 genes were differentially expressed specifically for Exacerbated_M2 samples, 87 genes in Exacerbated_M4_Viral and 108 genes in Exacerbated_M4_Unknown (Figure 2a).

### 2.3. Identification of Biological Processes

**Figure 3:**
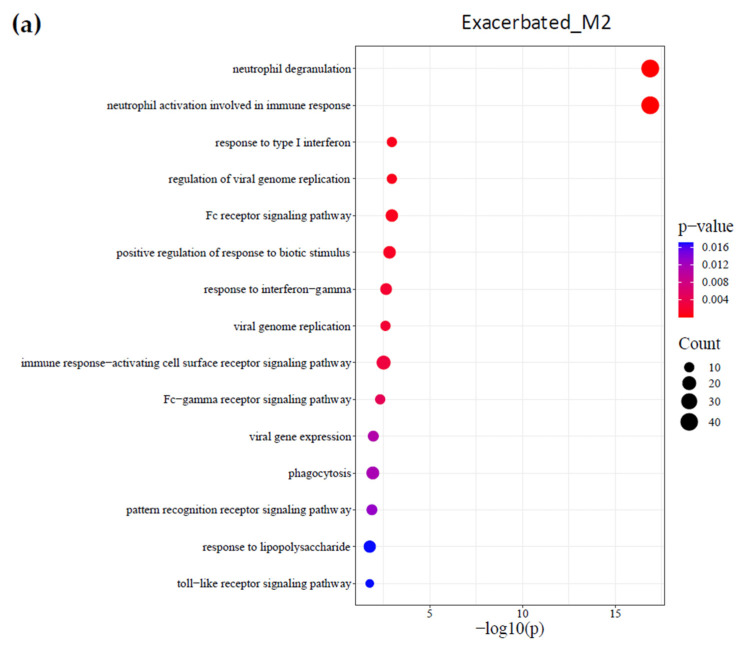

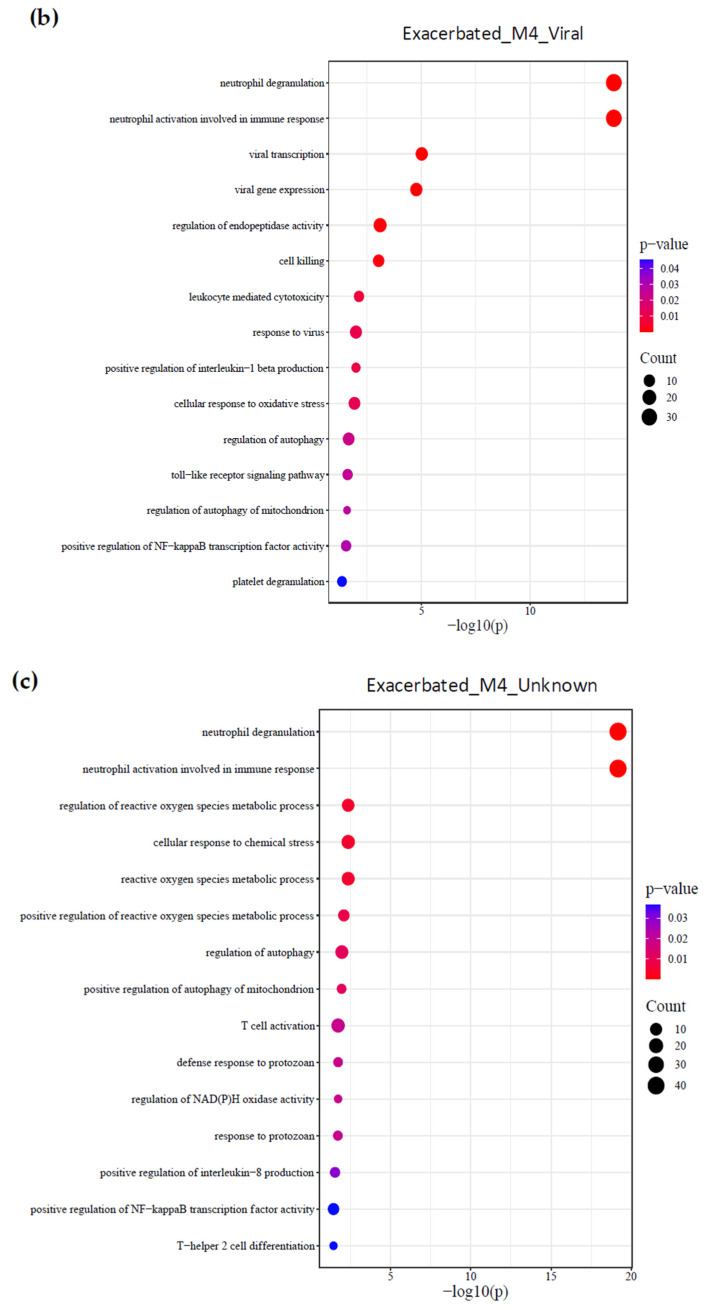
Enrichment pathway analysis of significant genes across clinical groups. Enrichment bubble plot showing the top enriched pathways identified for significant genes in each clinical group: Exacerbated_M2 (**a**), Exacerbated_M4_Viral (**b**), and Exacerbated_M4_Unknown (**c**). Biological processes were identified using Gene Ontology and ordered by statistical significance, with pathways meeting a false discovery rate (FDR) cutoff of <0.05 considered significant. The length of each bar represents the enrichment score, indicating the proportion of significant genes involved in each pathway.

To identify key biological processes related to the pathophysiology of each type of exacerbation, a pathway enrichment analysis was performed. In all exacerbated groups, neutrophils were significantly activated [neutrophil activation involved in immune response and neutrophil degranulation (*p*-value ≤ 0.05)]. In samples classified as exacerbated by bacterial origin (Exacerbated_M2), 142 biological pathways were identified as statistically significantly involved in the exacerbation. Among them, pathways related to receptor-mediated phagocytosis [phagocytosis, Fc signaling receptor pathway, Fc-gamma receptor signaling pathway, response to lipopolysaccharide, pattern recognition receptor signaling pathway, toll-like receptor signaling pathway, and immune response-activating cell surface receptor signaling pathway] and the antiviral response [response to type I interferon, viral genome replication, the regulation of viral genome replication, and response to interferon-gamma and viral gene expression] (Figure 3a).

In viral exacerbation samples (Exacerbated_M4_Viral), 107 biological processes were significantly activated. Among them, biological processes related to viral infection [viral transcription, viral gene expression, response to virus, and the regulation of endopeptidase activity], pro-inflammatory response [the positive regulation of NF-kappaB transcription factor activity, the positive regulation of interleukin-1 beta production, and platelet degranulation], innate and adaptive cytotoxic responses [cell killing and leukocyte-mediated cytotoxicity], and mitochondrial damage [the regulation of mitochondrial autophagy, the regulation of autophagy, and cellular response to oxidative stress] (Figure 3b).

Unknown exacerbations (Exacerbated_M4_Unknown) significantly activated 72 pathways. Among these, notable pathways included those associated with protozoan infection [response to protozoans and defense response to protozoans, the regulation of reactive oxygen species (ROS) metabolic processes, the positive regulation of ROS metabolic processes, and the regulation of NAD(P)H oxidase activity], mitochondrial damage [the regulation of autophagy and the positive regulation of mitochondrial autophagy], and asthma [T-helper 2 cell differentiation, the regulation of ROS metabolic processes, cellular response to chemical stress, the positive regulation of NF-kappaB transcription factor activity, and the positive regulation of interleukin-8 production] (Figure 3c).

### 2.4. Identification of Biomarkers

**Figure 4:**
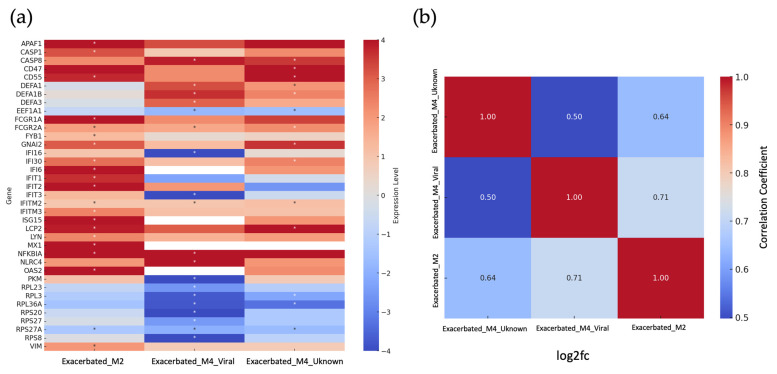
Heatmap analysis of gene expression and correlation analysis in COPD exacerbations. (**a**) Gene expression levels in the blood transcriptome following exacerbations in COPD patients, stratified by clinical groups (Exacerbated_M2, Exacerbated_M4_Viral, and Exacerbated_M4_Unknown). Differential expression of key genes across groups is represented. Statistical differences against stable samples *p* < 0.05 are identified with an asterisk (*). (**b**) Correlation analysis of gene expression patterns between the different clinical groups (Exacerbated_M2, Exacerbated_M4_Viral, and Exacerbated_M4_Unknown). The heatmap shows the correlation coefficients between groups, with higher values indicating greater similarity in immune responses.

To identify biomarkers for the diagnosis of each type of exacerbation, the differential expression (exacerbated vs. stable) of the genes involved in the biological processes shown in Figure 3 was analyzed. A total of 13 genes were identified as biomarkers for Exacerbated_M2 clinical group, as their statistically significant overexpression appeared exclusively in the bacterial-origin group. Interestingly, 7 of these 13 genes are part of the interferon-induced antiviral response: *IFITM3*, *IFIT1* and *IFIT2*, *OAS2*, *ISG15*, *MX1*, and *IFI6*. The other six genes encode proteins involved in the activation and migration of immune cells to the site of inflammation. These genes are as follows: *APAF1*, *CASP1*, *Fc gamma receptor Ia*, *FYB1*, *LYN*, and *VIM* (Figure 4a, Exacerbated_M2 column).

In the case of Exacerbated_M4_Viral clinical group, nine genes were identified as biomarkers. Two of them (*DEFA3* and *NLRC4*) were significantly overexpressed only in this clinical group. The other seven genes were significantly down-regulated only in this exacerbation type (*IFI16*, *IFIT3*, *PKM*, *RPL23*, *RPS20*, *RPS27*, and *RPS8*). Interestingly, these genes have complementary functions; two of them (*IFIT3* and *IFI16*) are involved in viral defense, and the other five genes (*PKM*, *RPL23*, *RPS20*, *RPS27*, and *RPS8*) play roles in the regulation of protein synthesis, which is crucial for the cellular stress response, including immunological stress (Figure 4a, Exacerbated_M4_Viral column).

In the Exacerbated_M4_Unknown group, only *CD47* was identified as a biomarker (Figure 4a, unknown column).

The immune responses observed in the unknown group show more similarity to the Exacerbated_M4_Viral group (correlation 0.71) than to the Exacerbated_M2 group (correlation 0.64) (Figure 4b).

## 3. Discussion

This study provides preliminary insights into potential mechanisms and genes associated with exacerbations in COPD patients, which warrant further validation in larger cohorts, making a transcriptomic comparison across different clinical groups based on etiology (bacterial, viral, or unknown). Differential expression analysis and pathway enrichment of the blood transcriptome have identified key biomarkers and biological processes that differentiate the various types of exacerbations. These results raise new hypotheses about the etiologies of exacerbations and identify new targets to develop treatments and improve the prevention of exacerbations.

Neutrophils remain central to the pathology of COPD exacerbations. Regardless of the causative agent of the exacerbation, biological processes involved in neutrophil activation (like neutrophil activation involved in the immune response and neutrophil degranulation) were significantly activated. In addition, median BNC was elevated (>6000 cells/µL) [10], and an NLR higher than 3.5 is considered a biomarker of acute COPD exacerbation [11,12]. Although it is well known that an increase in neutrophils is a hallmark of exacerbations, attacking neutrophils directly could be counterproductive because not only is inflammation reduced, but defense against pathogens is also reduced [13]. For this reason, elucidating the downstream pathways activated by neutrophils is extremely important. A previous study using transcriptomics has shown that COPD exacerbations significantly modulate pathways such as PI3K-AKT, MAPK, and NF-κB, which are associated with both inflammatory processes and immune responses [14]. Furthermore, co-expressed gene modules associated with FEV1 (Forced Expiratory Volume in 1 second) were identified, highlighting the biological relevance of these clusters in lung function [15]. However, current studies are limited and do not stratify patients based on the cause of exacerbations, hindering progress toward personalized medicine. Additionally, more studies are needed to discriminate the inflammatory response of COPD exacerbation from asthma [16].

According to our results, bacterial-induced exacerbation significantly activates phagocytosis. Considering that phagocytosis is the first mechanism neutrophils use to attack bacteria, and that both the Fc receptor pathway and the toll-like receptor signaling pathway are activated, our hypothesis is that activated neutrophils may detect opsonized bacteria through Fc receptors or un-opsonized bacteria via TLR receptors [17].

In the case of virus-induced exacerbation, the increased expression levels of *DEFA3* may suggest a potential increase in azurophilic neutrophil granules, as *DEFA3* belongs to the α-defensins family, which constitutes approximately 50% of the protein content in these granules [18]. In line with this, in this clinical group, there is significant regulation of endopeptidase activity, suggesting up-regulation of neutrophil serine proteases, such as neutrophil elastase, the third major component of azurophilic granules, which plays a key role in the processing of α-defensins [17]. Our hypothesis is that the pro-inflammatory response observed in this clinical group is a consequence of proteins released from azurophilic granules, which activate surrounding platelet degranulation, leading to the secretion of pro-inflammatory cytokines and chemokines [18]. Furthermore, this process could be associated with the formation of neutrophil extracellular traps (NETs), as the release of proteins like neutrophil elastase and α-defensins has been shown to play a key role in NET formation, amplifying the inflammatory response [13,19].

In the context of exacerbations of unknown origin, neutrophils appear to play a multifaceted role. The activation of biological processes related to protozoan defense suggests that neutrophils may engage in a respiratory burst, a key process mediated by the enzyme NAD(P)H oxidase, which generates reactive oxygen species (ROS) [20]. These ROS not only have microbicidal functions but can also amplify local inflammation. To ensure efficient neutrophil function against protozoan infections, neutrophils probably activate mitophagy to reduce the production of additional ROS [21].

Furthermore, patients experiencing these exacerbations seem to be in a context of neutrophilic asthma, which is more commonly associated with respiratory infections or exposure to irritant chemicals rather than with allergies, as these patients do not show eosinophilia (the median BEO was 110 cells/µL) [22]. The presence of asthma together with the activation of pathways against protozoan infection suggest that the Exacerbated_M4_Unknown group is actually composed of patients with ACOS (asthma–COPD overlap syndrome) [23]. Other papers have previously described the significantly higher incidence of protozoans in patients with asthma compared with COPD patients (58.8% vs. 26.6%, *p* = 0.046) [24], but also, in at least 50% of the patients with acute exacerbations of respiratory symptoms (including COPD patients), the presence of protozoa has been identified in sputum [6]. In addition, it describes a significant association between COPD exacerbation and intestinal protozoa [25]. However, further investigations are needed to elucidate if protozoans are the causative agent of the exacerbations in this group of patients that could be treated with anti-protozoan medications like metronidazole.

Additionally, we have identified *CD47* as a potentially relevant biomarker for exacerbations of unknown origin. This finding provides a diagnostic tool for this clinical group. The role of *CD47* could be even more important given its function in inhibiting the phagocytosis of the protozoan with the “do not eat me” signal [26], suggesting that this biomarker might also have therapeutic implications in controlling inflammation. Therefore, confirming the utility of *CD47* in this context could represent a significant advancement in the personalized treatment of these groups of patients and open new avenues for targeted therapeutic strategies.

Although neutrophils play an important role in COPD exacerbations, other inflammatory mechanism are also involved. For instance, in these patients, *IFN* activation may be delayed compared to healthy individuals [27]. To compensate for this alteration, alternative defense mechanisms are likely up-regulated [28]. One such compensatory mechanism is the heightened activation of *DEFA3*, which plays a critical role in immune defense or the pro-inflammatory response (mediated by NF-kappaB), which may exacerbate inflammation rather than effectively control viral infection [29]. For this reason, enhancing *IFN* responses could be beneficial for patients with COPD [30]. In fact, clinical studies have indicated that inhaled recombinant IFN-β may be effective in up-regulating viral defense to accelerate viral clearance and reduce the proportion of patients with bacterial secondary infections [31].

In virus-induced exacerbations, while respiratory viruses can be detected using multiplex PCR, a robust *IFN* response is notably absent. Instead, only biological processes associated with the viral life cycle (viral transcription, viral gene expression, and response to virus) are activated. Conversely, in exacerbations classified as bacterial, respiratory viruses are not detected; however, an IFN response is observed, with biological processes such as response to type I interferon, the regulation of viral genome replication, and response to interferon-gamma being activated. These findings suggest that a prior viral infection may predispose patients to a secondary bacterial exacerbation [32].

This pattern indicates that both viral and bacterial exacerbations could ultimately be initiated by an initial viral infection, creating a cellular environment characterized by a constitutive antiviral state in bacterial exacerbations.

The findings presented in this study could represent a significant advancement in the management of exacerbations in patients with COPD. However, an important limitation of this study is the inability to confirm the presence of protozoa in the sputum of the patients, as the samples were not processed in the laboratory in a way suitable for protozoa identification, either by microscopy or molecular techniques. Future studies should address this limitation to identify and confirm the presence of protozoa in exacerbated COPD populations. Additional limitations must also be acknowledged. Due to the small sample size, these results should be considered preliminary and require independent validation in larger cohorts. Furthermore, this study was conducted in a single center, which restricted the inclusion of mild cases, limiting the generalizability of the results. Moreover, potential confounding factors, such as comorbidities and medication use, were not fully accounted for in this study and could affect the generalizability and interpretation of the results.

The findings presented in this study could represent a significant advancement in the management of exacerbations in patients with COPD.

## 4. Materials and Methods

### 4.1. Patient Selection

This is a prospective study involving 16 patients with a confirmed diagnosis of COPD. Each patient provided a blood sample (*n* = 16) and a sputum sample (*n* = 16) during exacerbated states. Additionally, the same samples were collected from 5 volunteer patients in a stable state one month after overcoming the exacerbation and following hospital discharge; the samples were collected throughout 2019. Sputum samples taken from patients during exacerbation were used to classify patients based on the etiology of their exacerbation (viral-infection-related exacerbations, bacterial-infection-related exacerbations, and exacerbations of unknown cause). Classification was performed through a microbiota analysis as previously described in [9]. In this case, we used an enhanced version of this classification by including the identification of respiratory viruses through the Filmarray Pneumonia panel (Biomerieux, Marcy-l’Étoile, France), which allowed for differentiation between patients with viral-infection-related exacerbations and those with exacerbations of unknown cause. This study was approved by the ethics committee of the General University Hospital of Elche (PI37/2017), and all patients provided written informed consent.

### 4.2. Blood Transcriptomic Analysis

Blood samples were preserved in Tempus Blood DNA preservation tubes (ThermoFisher, Waltham, MA, USA) to ensure nucleic acid stability. Total RNA extraction from blood samples was performed using a Spin RNA Isolation Kit (ThermoFisher), following the manufacturer’s instructions. Subsequently, cDNA libraries were prepared for sequencing on the Oxford Nanopore MinION platform using a Ligation Sequencing (SQK-LSK109) Kit in combination with a Direct cDNA Sequencing Kit (SQK-DCS109) (Oxford, UK).

Bioinformatic analysis of the transcriptome was conducted with a wf-transcriptome pipeline from the EPI2ME Labs package v1.6.1 (Nanopore, Oxford, UK). This pipeline included read mapping, gene identification and quantification, and differential gene expression analysis, allowing for the identification of key biological processes associated with exacerbations.

The results of the transcriptomic analysis based on Gene Ontology pathway enrichment were visualized using the SRPlot platform [33], enabling a comparison of gene expression profiles and the identification of biological processes across different clinical groups.

### 4.3. Statistical Analysis

Differences in participants’ characteristics between basal and exacerbated status were assessed with Chi-square and using Student’s t-test for continuous variables. Significant differences were considered when the *p*-value was <0.05. Statistical analyses of differential gene expression for each group of blood samples from exacerbated patients were conducted using samples from the stable group as the control. These analyses were performed using the wf-transcriptome analysis package, employing the “Differential gene expression” script aggregated for the clinical samples in each group (viral, bacterial, or unknown exacerbation). Transcript quantification of the genes was conducted using the Salmon package v1.10.1, which utilizes a *quasi-mapping* approach to efficiently align reads to the reference transcriptome, applying bias corrections for fragment GC content and length distribution to enhance accuracy. Differential expression of the genes identified in each clinical group was calculated using edgeR v4.0, employing negative binomial generalized linear models and the Trimmed Mean of M-values (TMM) normalization method to account for library size and composition differences. Likelihood ratio tests were used to identify differentially expressed genes (DEGs), with statistical significance set at FDR-adjusted *p*-values < 0.05 and a |logFC| > 1. Subsequent pathway enrichment analyses incorporated DEGs to identify biological processes and overexpressed pathways, applying corrections for multiple comparisons. Additionally, protein clustering analyses were performed using STRING (https://string-db.org), enabling the identification of protein–protein interaction networks and functional clusters associated with each clinical group.

## 5. Conclusions

This study provides preliminary insights into the molecular mechanisms underlying COPD exacerbations, highlighting neutrophils as central players across bacterial, viral, and unknown etiologies. Bacterial exacerbations are marked by phagocytic activity, viral exacerbations involve DEFA3 and inflammatory pathways, and unknown etiologies suggest protozoan involvement and possible overlap with asthma–COPD overlap syndrome (ACOS). Immune disruptions, such as delayed interferon responses, further contribute to inflammation, emphasizing the need for targeted therapies. These findings suggest new hypotheses and therapeutic targets.

## Figures and Tables

**Table 1:** Participant’s characteristics in basal and exacerbated status.

Characteristics	Stables (*n* = 5)	Exacerbated (*n* = 16)	*p*-Value
**Age (years, median, IQR)**	76.0 (67.0–79.0)	74.0 (67.75–79.25)	0.736
**Sex at birth**			
Male	5 (100.00%)	13 (81.25%)	0.754
Female	0 (0%)	3 (18.75%)	
**GOLD**			
1E	0 (0%)	1 (6.25%)	0.758
2E	0 (0%)	2 (12.5%)	
3E	3 (60.00%)	7 (43.75%)	
4E	2 (40.00%)	5 (31.25%)	
**Microbiome**			
Stable or unknown exacerbated (M4)	5 (100%)	4 (25.0%)	0.016
Viral exacerbated (M4 + respiratory virus)		4 (25.0%)	
Bacterial exacerbated (M2)		8 (50.0%)	
**Cell count (cells/µL, median, IQR)**			
Leukocytes		8825 (7973–9443)	
Neutrophils		6245 (4695–6918)	
Lymphocytes		1275 (758–1965)	
Eosinophils		135 (65–258)	

**Table 2:** Shared genes between the three clinical groups.

Gene Name	Description
*AC010970.1*	Long intergenic non-protein coding RNA
*ANXA3*	Annexin A3
*ARID4B*	AT-rich interaction domain 4B
*CLEC4E*	C-type lectin domain family 4 member E
*DYNC1LI1*	Dynein cytoplasmic 1 light intermediate chain 1
*ELP3*	Elongator acetyltransferase complex subunit 3
*FCGR2A*	Fc fragment of IgG receptor IIa
*FKBP5*	FK506 binding protein 5
*FLOT2*	Flotillin 2
*GCA*	Grancalcin
*HCK*	Hematopoietic cell kinase
*HDLBP*	High-density lipoprotein binding protein
*IFITM2*	Interferon-induced transmembrane protein 2
*JPT1*	Jupiter microtubule-associated homolog 1
*KCNJ2*	Potassium inwardly rectifying channel
*LUC7L2*	LUC7-like protein 2
*MAPKAP1*	Mitogen-activated protein kinase associated protein 1
*MATR3*	Matrin 3
*MED23*	Mediator complex subunit 23
*PRR13*	Proline-rich protein 13
*RAD23B*	RAD23 homolog B
*RASGRP4*	RAS guanyl-releasing protein 4
*RBM47*	RNA-binding motif protein 47
*RCBTB2*	Regulator of chromosome condensation and BTB domain containing 2
*RPS27A*	Ribosomal protein S27a
*S100A8*	S100 calcium-binding protein A8
*S100A9*	S100 calcium-binding protein A9
*SH3GLB1*	SH3 domain-containing protein L-binding protein 1
*SRPK1*	Serine/arginine-rich protein kinase 1
*STOM*	Stomatin
*STXBP2*	Syntaxin binding protein 2
*TLR8*	Toll-like receptor 8
*TTN*	Titin
*VPS72*	Vacuolar protein sorting 72
*ZNF512*	Zinc finger protein 512

## Data Availability

The original contributions presented in this study are included in the article and Supplementary Materials. Further inquiries can be directed to the corresponding author.

## Supplementary Materials

The following supporting information can be downloaded at: https://www.mdpi.com/article/10.3390/ijms26020627/s1.

## Abbreviations

The following abbreviations are used in this manuscript:

COPD	Chronic obstructive pulmonary disease
rRNA	Ribosomal Ribonucleic Acid
PCR	Polymerase Chain Reaction
IFITM3	Interferon-Induced Transmembrane Protein 3
ISG15	Interferon-Stimulated Gene 15
DEFA3	Defensin Alpha 3
CD47	Cluster of Differentiation 47
ROS	Reactive oxygen species
NAD(P)H	Nicotinamide Adenine Dinucleotide Phosphate Hydrogen
ACOS	Asthma–COPD overlap syndrome
NLR	Neutrophil to lymphocyte ratio
IFN	Interferon
NET	Neutrophil extracellular trap
BNC	Blood neutrophil count
BEO	Blood eosinophil count
IQR	Interquartile range
GOLD	Global Initiative for Chronic Obstructive Lung Disease
FDR	False discovery rate
cDNA	Complementary DNA
MinION	Oxford Nanopore MinION Sequencer
