# Supplementary material for: Gene Expression Profiles Reveal Distinct Mechanisms Driving Chronic Obstructive Pulmonary Disease Exacerbations
Source: Int J Mol Sci. 2025 Jan 13;26(2):627. doi: 10.3390/ijms26020627 (PMC11765561; doi:10.3390/ijms26020627)
Supplement: Supplementary file 1 [file ijms-26-00627-s001.zip › Figure_S1.pdf]

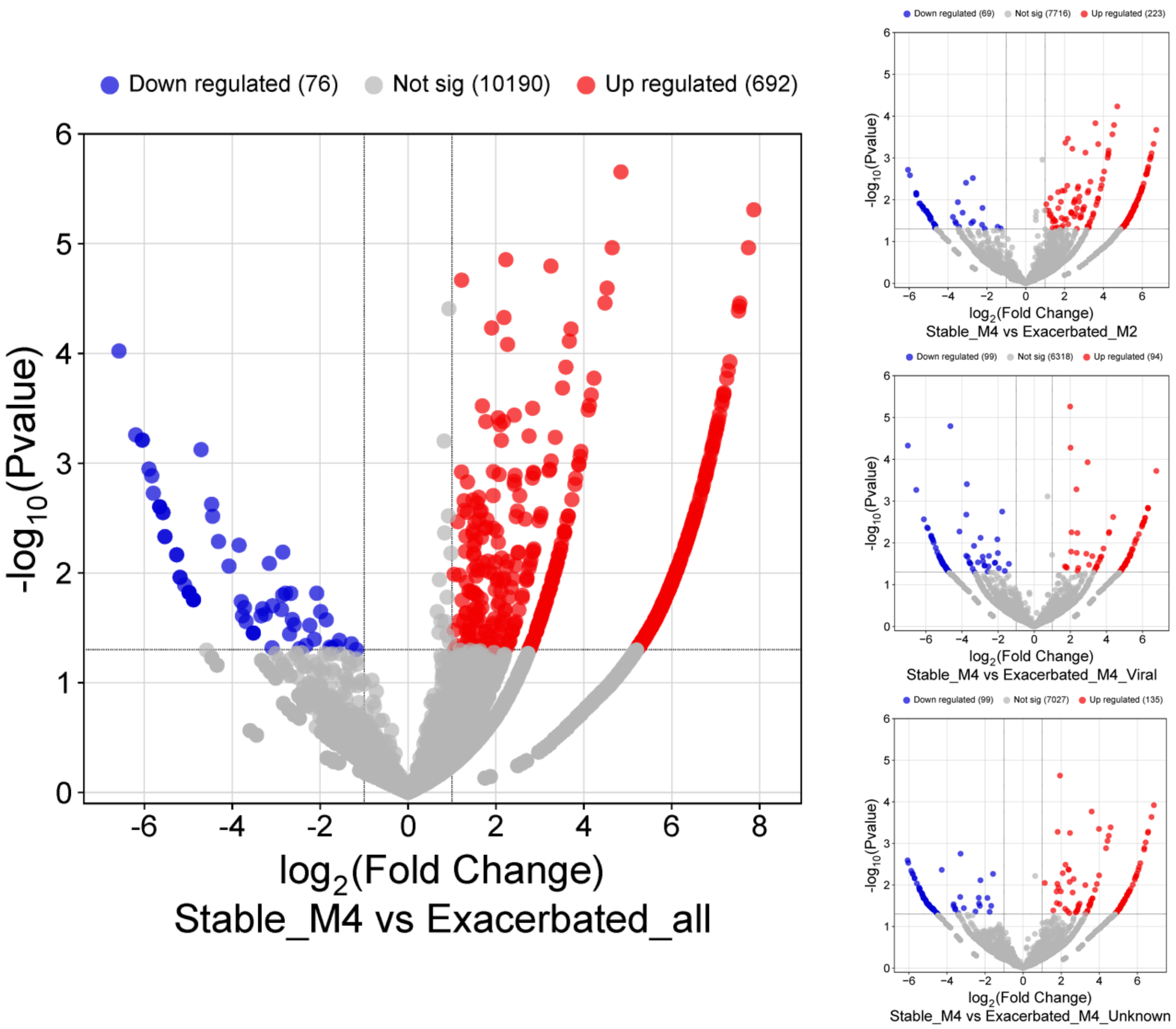

Figure S1. Identification of genes with modified expression during exacerbation in COPD patients. Volcano plot of COPD-related DEGs, graphed with log2 (Fold Change) on the abscissa and –log10 or the ordinate (adjusted P value). Nodes in red represent up-regulated genes, nodes in gray represent genes not significantly differentially expressed and nodes in blue represent genes down-regulated. The volcano plot was broken down into three to observe the differences between the different clinical groups.
